# Aberrant brain functional networks in type 2 diabetes mellitus: A graph theoretical and support-vector machine approach

**DOI:** 10.3389/fnhum.2022.974094

**Published:** 2022-10-12

**Authors:** Lin Lin, Jindi Zhang, Yutong Liu, Xinyu Hao, Jing Shen, Yang Yu, Huashuai Xu, Fengyu Cong, Huanjie Li, Jianlin Wu

**Affiliations:** ^1^Graduate School, Tianjin Medical University, Tianjin, China; ^2^Department of Radiology, Affiliated Zhongshan Hospital of Dalian University, Dalian, China; ^3^School of Biomedical Engineering, Dalian University of Technology, Dalian, China; ^4^Faculty of Information Technology, University of Jyvaskyla, Jyvaskyla, Finland; ^5^Department of Endocrinology, Affiliated Zhongshan Hospital of Dalian University, Dalian, China

**Keywords:** type 2 diabetes mellitus, cognitive function, auditory cortex, resting-state MRI, support vector machine, topological properties

## Abstract

**Objective:**

Type 2 diabetes mellitus (T2DM) is a high risk of cognitive decline and dementia, but the underlying mechanisms are not yet clearly understood. This study aimed to explore the functional connectivity (FC) and topological properties among whole brain networks and correlations with impaired cognition and distinguish T2DM from healthy controls (HC) to identify potential biomarkers for cognition abnormalities.

**Methods:**

A total of 80 T2DM and 55 well-matched HC were recruited in this study. Subjects’ clinical data, neuropsychological tests and resting-state functional magnetic resonance imaging data were acquired. Whole-brain network FC were mapped, the topological characteristics were analyzed using a graph-theoretic approach, the FC and topological characteristics of the network were compared between T2DM and HC using a general linear model, and correlations between networks and clinical and cognitive characteristics were identified. The support vector machine (SVM) model was used to identify differences between T2DM and HC.

**Results:**

In patients with T2DM, FC was higher in two core regions [precuneus/posterior cingulated cortex (PCC)_1 and later prefrontal cortex_1] in the default mode network and lower in bilateral superior parietal lobes (within dorsal attention network), and decreased between the right medial frontal cortex and left auditory cortex. The FC of the right frontal medial-left auditory cortex was positively correlated with the Montreal Cognitive Assessment scales and negatively correlated with the blood glucose levels. Long-range connectivity between bilateral auditory cortex was missing in the T2DM. The nodal degree centrality and efficiency of PCC were higher in T2DM than in HC (*P* < 0.005). The nodal degree centrality in the PCC in the SVM model was 97.56% accurate in distinguishing T2DM patients from HC, demonstrating the reliability of the prediction model.

**Conclusion:**

Functional abnormalities in the auditory cortex in T2DM may be related to cognitive impairment, such as memory and attention, and nodal degree centrality in the PCC might serve as a potential neuroimaging biomarker to predict and identify T2DM.

## Introduction

Diabetes mellitus is a complex disease involving multiple systems and damage to multiple organs. Type 2 diabetes mellitus (T2DM) is the most common type of diabetes, accounting for 90–95% of all cases (Xie and Burke, 2020), and it is a well-recognized risk factor for cognitive decline and dementia (Livingston et al., 2020; Nguyen et al., 2020). Notably, even in the early stages of T2DM, various of cognitive impairments can occur with respect to attention, memory, vision, information processing speed, and executive ability (Kim, 2019), but the underlying neural mechanisms of these impairments are unclear.

Functional magnetic resonance imaging (fMRI) techniques are robust tools for studying the mechanism of brain function and disease. Several studies have applied fMRI to reveal the mechanism of brain function impairment. The dysfunction of T2DM is widely studied in default mode network (DMN), which is primarily involved in normal cognitive functioning, such as memory processing, executive function and self-referencing. It is metabolically active and a site of increased aerobic glycolysis, making itself susceptible to amyloid accumulation and altered glucose metabolism inherent in diabetes (Buckner et al., 2005). Impaired functional activity of DMN has been reported in previous studies, Cui et al. (2015) showed that the connectivity of dissociated pattern of DMN in T2DM was associated with impaired cognition. Qi et al. (2017) revealed that T2DM performed poorly in episodic memory and showed aberrant DMN functional connectivity (FC). Some studies (Xia et al., 2015; Yang et al., 2016; Lei et al., 2021) showed impaired FC in the ventral and dorsal attention network (DAN) was associated with cognitive dysfunction in T2DM, such as attention, executive function, and processing speed.

In addition to impaired FC, several studies have explored the topological properties of the large-scale network are explored in T2DM. The graph theory analysis can effectively explore the changes in the topology of whole-brain network features, such as small-world properties, modular organization, and hub nodes. This method has been used to explore whole-brain function in subjects with T2DM, but the results are not consistent. For example, Van Bussel et al. (2016) found that T2DM and pre-T2DM have significantly higher normalized cluster coefficients and a higher global and local efficiency in the whole brain regions than healthy controls (HC), a high global efficiency was associated with reduced processing speed. Chen et al. (2017) and Qin et al. (2019) demonstrated that compared to HC, T2DM with normal cognitive function showed a higher small-worldness, decreased global efficiency, a higher local efficiency, reduced and increased nodal characteristics (degree, efficiency, and between) in the frontal, parietal, temporal, and occipital lobes. Xu et al. (2019) showed that the small-world characteristics between the T2DM and HC did not differ markedly; the T2DM had an increased nodal degree and efficiency mainly located in the right inferior temporal gyrus. Xiong et al. (2020) showed that compared to T2DM with normal cognition function, T2DM with cognitive impairment group had a higher clustering coefficient and local efficiency, as well as increased nodal properties (efficiency, degree, and betweenness) in the parietal, temporal, and occipital lobes, but decreased in the right inferior temporal gyrus. On the other hands, according to Zhou et al. (2022), there were no significant differences in the global and nodal properties between the T2DM patients with and without cognitive impairment. Briefly, the complex alterations in the topological properties of functional brain networks in patients with T2DM are uncertain, and their correlation to cognitive function is unclear. Two of the main factors underlying these inconsistent results include a small sample size and rough brain parcelation.

In the present study, we utilized large sample size by merging fMRI data from two datasets and fine brain network template to explore the brain network changes in patients with T2DM based on FC and graph theory methods, and analyzed the correlation with both clinical indicators and cognitive scores to explore the potential neural mechanisms of cognitive impairment caused by T2DM. Furthermore, we evaluated and explored the potential neuroimaging biomarker that might be valuable in predicting and diagnosis T2DM.

## Materials and methods

### Subjects

In this study, 80 patients with diabetes mellitus and 55 healthy subjects matched for gender, age, and years of education were recruited from the Affiliated Zhongshan Hospital of Dalian University. All subjects were right-handed and able to complete magnetic resonance imaging (MRI) examinations and cognitive function tests. They were informed of the specific content of the study before obtaining, signed informed consent.

Standardized diagnoses and assessments were carried out for all the participants with respect to a medical history interview, essential laboratory tests, an MRI examination of the brain, and neuropsychological scale tests. The diagnostic criteria for T2DM were based on the 2014 American Diabetes Association guidelines. In the case of a fasting glucose level >6.1 mmol/L or a postprandial glucose level >7.8 mmol/L, the control participants were excluded from this study. None of participants had any contraindications to MRI examination, were not drug-, alcohol-, or tobacco-dependent, and had not suffered from any traumatic brain injury or brain lesions, such as cerebral infarction or tumor, or neurological or mental diseases unrelated to T2DM.

### Cognitive scale test

The Montreal Cognitive Assessment scale (MoCA) was used to evaluate the cognitive function of the participants in terms of executive and visuospatial ability, memory, and other aspects. Those with scores ≥26 were considered to have normal cognitive function. Subsequently, the participants completed the Verbal Fluency Test (VFT), Auditory Verbal Memory Test (AVLT), Digit Span Test (DST), and Trail Making Test A (TMT-A) further examine specific aspects of the participants’ cognition function. The total duration of tests was approximately 40 min.

### Magnetic resonance imaging data acquisition

Magnetic resonance imaging data of 135 subjects from two different datasets were used in the present study. All data were collected using the same Siemens 3.0T Magnetom Verio MRI scanner with two different scanner parameters and the standard 12-channel head coil to obtain brain imaging data. During imaging, all participants closed their eyes, remained awake, and were placed in a supine position, with their heads tightly secured with foam pads and straps.

Dataset 1: A total of 44 patients with T2DM and 38 healthy controls (HC) were acquired from study 1. fMRI data were obtained using the following echo planar imaging (EPI) sequence: TR = 2,000 ms, TE = 30 ms, slice = 31, ST = 3.5 mm, FA = 90°, FOV = 240 mm × 240 mm, matrix = 64 × 64, 240 time points, and a scan time of 8 min 6 s. Whole brain three-dimensional (3D) structural T1 weighted imaging (T1WI) data were obtained using a 3D fast magnetization preparative gradient echo sequence (3D-MPRAGE). The scan parameters were as follows: TR = 2,530 ms, TE = 2.22 ms, slice = 192, ST = 1 mm, FA = 7°, MS = 224 × 224, FOV = 224 mm × 224 mm, VS = 1 mm × 1 mm × 1 mm, and a scan time of 5 min 28 s.

Dataset 2: A total of 37 patients with T2DM and 17 HCs were acquired from study 2. fMRI data were acquired with EPI, and the acquisition parameters were as follows: TR = 2,000 ms, TE = 30 ms, slice = 35, ST = 4 mm, FA = 90°, FOV = 240 mm × 240 mm, matrix = 64 × 64, 180 time points, and a scan time of 6 min 8 s. Whole brain 3D T1WI structural images were obtained using a 3D-MPRAGE. The scan parameters were as follows: TR = 1,900 ms, TE = 2.49 ms, slice = 176, ST = 1 mm, FA = 9°, MS = 224 × 224, FOV = 250 mm × 250 mm, matrix = 256 × 256, and a scan time of 4 min 26 s.

### Data processing

#### Functional connectivity analysis

The fMRI data was preprocessed based on FSL software, including scalp removal, removal of the first five times, temporal layer correction, head motion correction, spatial normalization to MNI space, and spatial smoothing. One T2DM subject from study 1 was excluded because of incomplete images. In order to maintain the same timepoints for fMRI data of all subjects, the first 175 time points of all the participants’ data were selected, such that their 4D data were consistent at 91 × 109 × 91 × 175. Then, we select a 2 mm × 2 mm × 2 mm brain template with 100 regions of interest (ROIs) designed by Thomas Yeo’s Laboratory^1^ which containing 17 brain networks (Schaefer et al., 2018) to calculate the FC matrix. For each subject, a 100 (regions) × 175 time-series matrix was obtained. The Pearson’s correlation coefficient was calculated for every two columns of the matrix. Two sample *t*-test of the correlation matrix was conducted based on a general linear model (GLM) to calculate the group difference, and the data acquisition effect, age and sex were included in GLM as covariates to be removed. The significant level was set to *P* < 0.0001 (uncorrected) to filter out differences in FC between the two groups.

#### Properties of brain networks based on graph theory

Based on the 135 correlation matrices formed by the functional connections in the previous step, GRETNA toolbox^2^ was used to construct a binary brain network by converting *Z*-matrix and assess the topological global and nodal properties of the brain functional network. The global properties included the characteristic path length (Lp), clustering coefficient (Cp), global efficiency (Eg), local efficiency (Eloc), the normalized clustering coefficient (γ), normalized characteristic path length (λ), small-worldness (σ = γ/λ > 1); the nodal parameters included node efficiency and node degree centrality. The sparsity threshold was set between 0.05 and 0.95 with a step size of 0.05. The area under the curve (AUC) of all topological properties was calculated over the sparsity threshold. Then, we added and averaged the correlation matrices of all subjects after taking the absolute values (threshold | *r*| > 0.85, 0.8, 0.7, and 0.6) to obtain the average brain matrix and comparison map.

#### Support vector machine classification analysis

Support vector machine (SVM) based on Python toolkit was applied to establish a prediction model and potential neuroimaging biomarker for predicting T2DM from HC. SVM has two major hyperparameters, the regularization coefficient (c) and the Gaussian kernel coefficient (gamma, g). For parameter optimization, c and g values are considered within a certain range. For a specific c and g, the training set is taken as the original data-set, and the K-CV method is used to obtain the classification accuracy of the validation set of the combination of each c and g set. Finally, the c and g group with the highest validation classification accuracy of the training set is considered as the best parameters. We used the grid parameter optimization method for parameter optimization. In this study, c was optimized in the range (2^–1^, 2^3^) and g in the range (2^–4^, 2^1^). The data-set was divided into a training and test set at a ratio of 8:2, and the accuracy of the prediction model was assessed. Different graph indicators (degree centrality and node efficiency) from different regions (all the region, group different regions) were used for training to establish the predictive model to get the best biomarkers to identify T2DM.

### Statistical analysis

SPSS software (version 22.0; Chicago, IL, USA) was used to compare the differences in demographics, clinical variables, and cognitive performance between the HC and T2DM groups using independent two-sample *t*-test, while chi-square tests were performed for proportions. Age, gender and MRI data acquisition effects were included as covariates for removal, the partial correlation analysis were conducted between FC and clinical/cognitive scores, the threshold was set to *P* < 0.05, Bonferroni correction was used to control the multiple comparisons. Bonferroni correction was applied to compare the differences of topological metrics between T2DM and HC; the threshold was set to *P* < 0.05.

## Results

### Demographic, clinical, and cognitive results

The demographic, clinical and neuropsychological information of all subjects was presented in Table 1. No significant differences were detected in gender, age, years of education, VFT, AVLT, and DST scores between the two groups in this study (*P* > 0.05). The T2DM patients had higher levels of fasting blood glucose, lower MoCA, AVLT5min, AVLT20min scores, and poor TMT-A scores than HC (*P* < 0.05 corrected with Bonferroni correction).

**TABLE 1:** Comparison of general dates between patients with T2DM and HC.

	T2DM	HC	*t*/*x*^2^	*P*
Age (years)	59.79 ± 5.36	58.09 ± 4.44	1.94	0.054
Gender (M/F)	37/43	22/33	0.26	0.613
Education (years)	10.97 ± 3.18	11.29 ± 2.72	−0.697	0.497
History (years)	10.70 ± 5.87	−	−	−
FPG (mmol/L)	12.21 ± 6.913	4.89 ± 0.44	15.849	<0.001*
MoCA	24.51 ± 3.00	26.74 ± 1.62	−5.004	<0.001*
VFT	21.50 ± 4.75	23.27 ± 6.47	−1.416	0.161
AVLT	23.74 ± 4.69	25.41 ± 3.70	−1.737	0.086
AVLT5min	9.16 ± 2.06	10.19 ± 1.52	−2.49	0.015*
AVLT20min	8.91 ± 2.40	10.03 ± 1.49	−2.020	0.043*
DST	11.41 ± 2.23	12.24 ± 2.45	−1.606	0.108
TMT_A	63.49 ± 28.06	50.63 ± 16.33	1.98	0.047*

*P < 0.05.

### Alterations in functional connectivity and correlations

**FIGURE 1:**
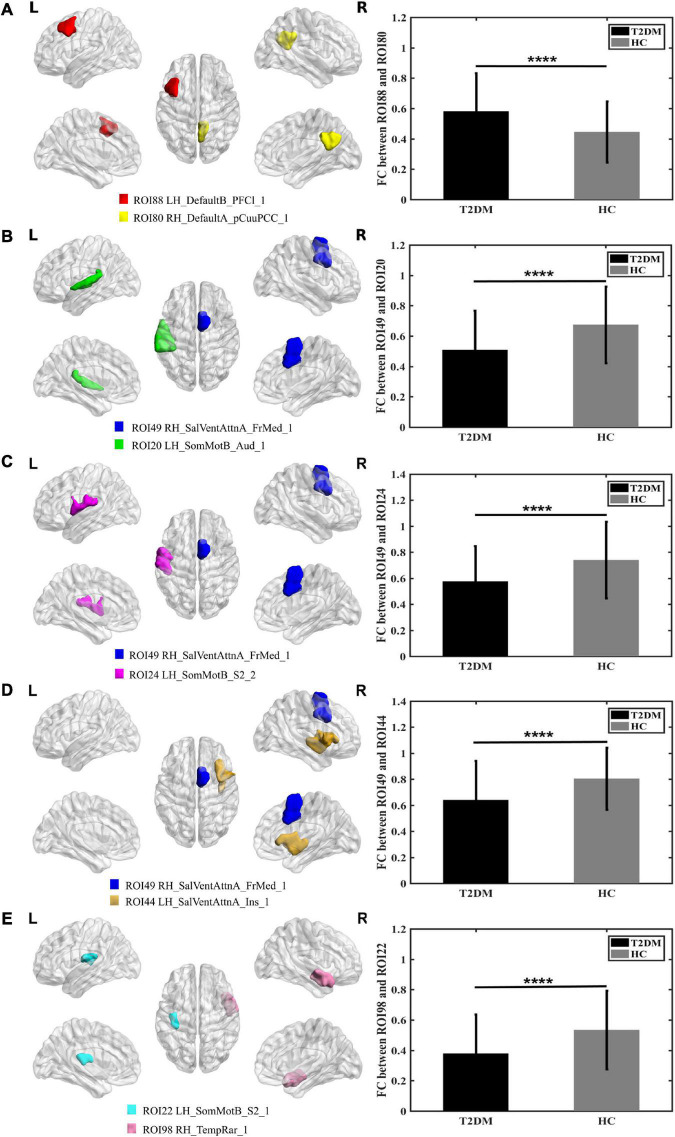
Graphs show that significant difference in the functional connectivity between T2DM and HC **(A–E)**. **(A)** The colored brain regions represent significantly increased functional connectivity in T2DM compared with HC; **(B–E)** the colored brain regions represent significantly decreased functional connectivity in T2DM compared with HC. ****P ≤ 0.001.

Figure 1A shows that compared to HC, the FC between the right DefaultA-precuneus/posterior cingulated cortex (PCC)_1 and the left DefaultB-lateral prefrontal cortex_1 was increased in patients with T2DM. Figure 1B shows that FC between right salience/ventral attention A-frontal medial_1 and the left sensorimotor B-auditory cortex_1 was decreased in patients with T2DM. Figure 1C shows that the FC between right salience/ventral attention A-frontal medial_1 and left sensorimotor B-S2 was decreased in patients with T2DM. Figure 1D shows the FC between right salience/ventral attention A-frontal medial_1 and the left salience/ventral attention A-insular was decreased in patients with T2DM. Figure 1E shows the FC between the left sensorimotor B-S2_1 and right TempRar_1 was decreased in patients with T2DM (^∗∗∗∗^*P* ≤ 0.001 uncorrected). These FC were positively correlated with MoCA, and negatively correlated with glucose for all participants (uncorrected), but no significant correlations were observed in T2DM, the more details of correlation analysis are placed in Supplementary material.

### Comparison and correlation analysis of topological properties of brain networks between patients with type 2 diabetes mellitus and healthy controls

**FIGURE 2:**
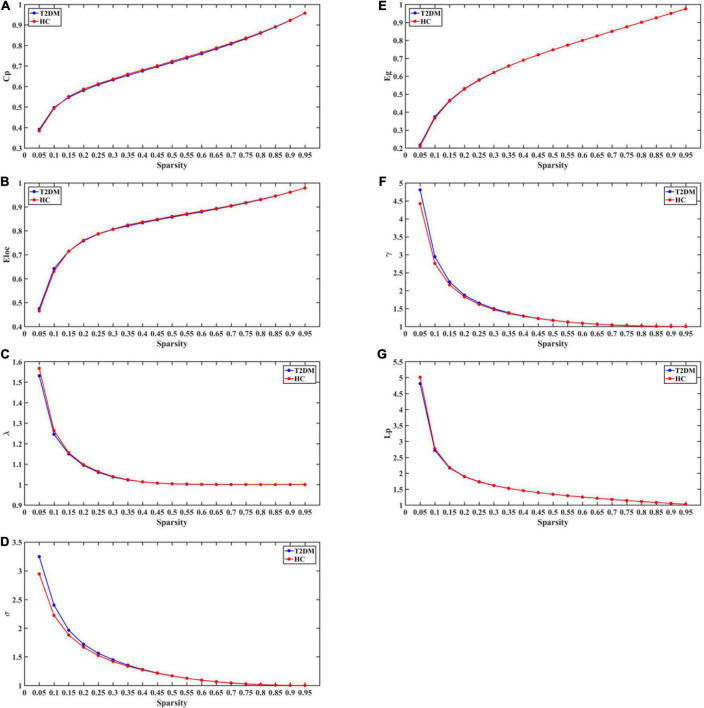
The global topological metrics of the whole-brain functional network of the T2DM and HC. Graph **(A–G)** show the small-word properties and network efficiency under different sparsity (0.05 < S < 0.95). Cp, clustering coefficient; Lp, characteristic path length; γ, normalized clustering coefficient; λ, normalized shortest path length; σ,small-world characteristics; Eg, global efficiency; Eloc, local efficiency.

Over the sparsity range 0.05–0.95, both groups exhibited similar small-world topological properties of high global and local efficiency, and no statistical differences were observed in Cp, Lp, Eg, Eloc, γ, λ, σ between the T2DM and HC groups (*P* > 0.05 Bonferroni corrected; see Figures 2A–G).

**FIGURE 3:**
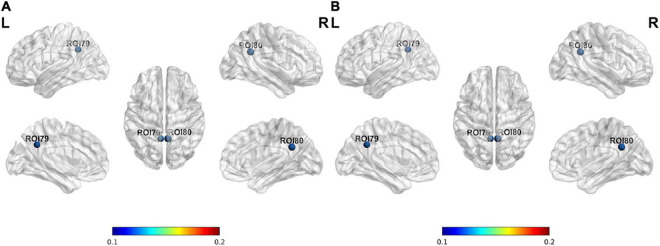
Differences in node properties between patients with T2DM and HC. **(A)** Nodal degree centrality; **(B)** nodal efficiency.

**FIGURE 4:**
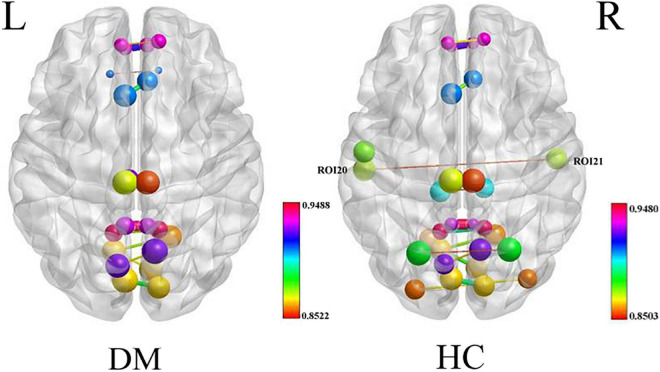
Average connectivity in the T2DM and HC groups, long-range connectivity between the bilateral auditory cortexes is missing in patients with T2DM.

The nodal degree centrality and efficiency of the bilateral pCunPCC_1 in T2DM group were higher than in HC group (Figures 3A,B and Table 2). No correlation was established between the topological properties with clinical and cognitive scores in T2DM group. Figure 4 shows long-range connectivity between the bilateral auditory cortexes was missing in T2DM compared to HC. Among the three additional thresholds, the long-range connectivity between the bilateral auditory cortexes was still missing (threshold 0.8) or was weak (threshold 0.7 and 0.6) in T2DM (the detail results for threshold *r* < 0.85 were shown in Supplementary material). In addition, lower average connectivity was detected in the bilateral visual network, DAN (bilateral superior parietal lobe_1), and Sal/VentAttn network in T2DM.

**TABLE 2:** Comparison of node properties differences between the T2DM and HC groups.

Brain regions		Nodal degree centrality		Nodal efficiency	
		*t*-Values	*P*-values	*t*-Values	*P*-values
T2DM > HC
ROI79	LH_DefaultA_pCunPCC_1	3.6789	0.00017	3.6625	0.00018
ROI80	RH_DefaultA_pCunPCC_1	4.0507	0.00004	4.0215	0.00005

### Support vector machine

**FIGURE 5:**
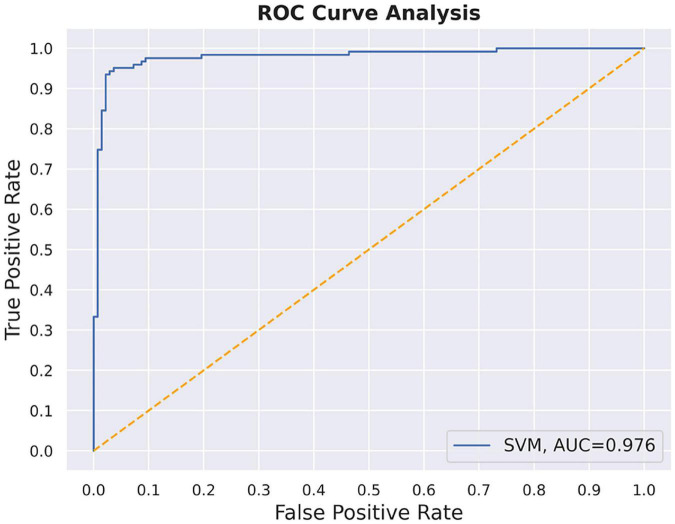
The ROC curves of the nodal degree centrality in PCC in the support vector machine model.

The SVM method was used to establish the prediction model with nodal degree centrality and efficiency. The accuracy of the method was 0.85 with all the group difference regions, which was lower than just using the nodal degree centrality of PCC; hence, PCC was used as the final classification feature. After parameter optimization, we finally selected the regularization coefficient “c” = 0.9259 and the kernel parameter “gamma” = 0.0625, the final model achieved 97.56% classification accuracy with the validation set (Figure 5).

## Discussion

In this study, by taking the advantages of larger sample size by merging MRI data from two datasets and fine brain parcelation with 100 ROIs, we find that T2DM shows: (1) increased connectivity between precuneus/PCC and later prefrontal cortex, and higher nodal properties of PCC; (2) decreased connectivity in the bilateral superior parietal lobes and decreased connectivity between right medial frontal cortex and left auditory cortex; (3) T2DM patients show functional decline in attention, delayed recall memory, and information processing speed, which is consistent with the results of previous studies (Qin et al., 2019; Li et al., 2021); (4) node degree centrality of PCC may be an effective and potential biomarker of T2DM.

### Type 2 diabetes mellitus shows increased connectivity between precuneus/posterior cingulated cortex and later prefrontal cortex, and higher nodal properties of posterior cingulated cortex

In the current study, we found that the FC between precuneus/PCC_1 and later prefrontal cortex_1 was increased (Figure 1A). Compared to HC, the node efficiency and node degree centrality of the bilateral precuneus/PCC were higher in patients with T2DM, which is consistent with the results of previous studies (Qin et al., 2019). The node efficiency represents the efficiency of the node in transmitting information to other nodes, and the node degree centrality reflects the importance of the node in the network (Zhang et al., 2021). Precuneus/PCC is the core node of the DMN, the impairment of these regions is frequently treated as the early signs of cognitive impairment (Leech and Sharp, 2014). The frontal lobe, more specifically the prefrontal cortex, has been historically associated with TMT due to its role in executive functions, such as attention, processing speed and planning, which are crucial for task coordination and completion (Talwar et al., 2020). In this study, TMT_A and MoCA scores were poorer in patients with T2DM, so the increased FC and higher nodal degree centrality and nodal efficiency of the bilateral PCC maybe recruit more nervous resources as a compensatory mechanism to maintain basic cognitive function, and reduce the process of cognitive decline in patients with T2DM.

Herein, SVM was employed to establish a prediction modal for classifying patients with T2DM and HC. The characteristic variable that finally entered the classification model was the PCC node degree centrality, and the classification accuracy rate reached 97.56%, indicating the reliability of the predictive model. Overall, these results might ascribe these factors as potential neuroimaging biomarkers for brain injury in patients with T2DM.

The MoCA test scores were lower in T2DM than in HC, the MoCA encompasses critical cognitive aspects, including memory, attention, and executive control. It can also detect mild cognitive impairment sensitively. Intriguingly, T2DM-related cognitive impairment may be an early stage of Alzheimer-related cognitive impairment (Liu et al., 2020); hence, this hyperconnectivity in T2DM patients may be an intermediate compensatory phase associated with FC changes or a sign of functional reorganization as a compensatory response to mild brain damage. Liu et al. (2019) demonstrated that FC of the intra-DMN had a U-like—rather than a simple linear—correlation with T2DM glucose metabolism and disease courses. In the early stage of the disease, the FC is enhanced, and as the blood glucose level rises further and the disease duration is prolonged, the FC begins to decrease. In addition, T2DM is strongly associated with depression, and changes in DMN FC (Tan et al., 2019). Specifically, the over-activity of DMN can be manifested as rumination, excessive internal self-focusing, and difficulties of switching from focusing on internal information processing to task-related processing, and this over activity may play a central role in depressive states (Tozzi et al., 2021).

### Type 2 diabetes mellitus shows decreased functional connectivity within bilateral superior parietal lobes

In the present study, we found decreased FC within bilateral superior parietal lobes (Figure 4), previous study showed that decreasing FA and increasing MD in bilateral parietal lobes in the T2DM, reflect a loss of white matter integrity (Tan et al., 2016; Moghaddam et al., 2019). Superior parietal lobes are involved in attention, and are core nodes of DAN, activation of the superior parietal lobes was correlated with attention switching ability in a distractor condition in the healthy people (Bledowski et al., 2004). This study showed that patients with T2DM spend more time on TMT-A test, which is highly sensitive to impaired visual search, attention, and psychomotor speed (Talwar et al., 2020). These results suggested that patients with T2DM have attention and neuromotor speed impairments, confirming the brain network functional changes. T2DM is a low level chronic inflammatory disease, accompanied by elevated IL-6 and C-reactive protein (CPR), while chronic inflammation can build a bridge between hyperglycemia and cognitive impairment (Cani et al., 2007; Zhao et al., 2012). Walker et al. (2020) demonstrated that increased levels of the plasma pro-inflammatory factor IL-6 were related to decreased FC in the DAN, and APOEε4 had a synergistic effect in promoting neurodegenerative changes, reducing the FC of the DAN, and increasing the risks of Alzheimer.

### Type 2 diabetes mellitus shows decreased connectivity between right medial frontal cortex and left auditory cortex, indicating hearing loss

This study found that the FC of right medial frontal cortex and left auditory cortex were decreased in patients with T2DM (Figure 1B). The graph theory studies demonstrated that long-range connectivity between the bilateral auditory cortexes is missing in patients with T2DM. The core region of salient/ventral attention network, the medial prefrontal lobe, is responsible for continuous information monitoring, participating in auditory attention and the early inhibitory modulation of inputs into the auditory cortex (Chen et al., 2018). A previous task-state MRI study (Araneda et al., 2018) suggested that executive function impairment caused by the prefrontal cortex may be a major factor in the generation and persistence of hearing impairments (tinnitus), as well as that cerebral blood flow (CBF) in this brain region was negatively correlated with TMT scores, indicating that abnormal perfusion in these brain regions is related to cognitive impairment. Taken together, these studies indicated that auditory function might be impaired in the T2DM. Nonetheless, hearing impairment is an under recognized complication of T2DM (Gupta et al., 2019; Li et al., 2020), and cochlear dysfunction and high-frequency hearing loss occur before the clinical symptoms of hearing impairment appear, once hearing impairment is diagnosed, treatment outcomes are poor. Furthermore, hearing impairment in T2DM may be due to chronic hyperglycemia affecting inner ear vascularization (Li et al., 2018), inner ear capillary wall thickening leading to impaired microcirculation, and the loss of outer hair cells in the lower and basal cochlea. Specifically, Xia et al. (2021) found that compared with patients without tinnitus, CBF of the auditory cortex and default network in patients with tinnitus was reduced, and diabetes exacerbated the decreased in cerebral perfusion. To date, only a few imaging studies have focused on functional changes in hearing-related brain regions in T2DM and their relation to cognition. Therefore, it is clinically significance to identify an appropriate method for detecting early hearing impairment in diabetic patients and examine its correlation with cognitive function to allow a timely development and implementation of preventative measures.

Nevertheless, this study also has some limitations. Firstly, though we tried to merging two datasets’ fMRI data to get a larger sample size, the sample size is still not large enough to get very robust and reliable results. Therefore, the current results need to be substantiated further using more multi-center fMRI data in the future. Secondly, the different treatment plans of T2DM patients may influence the results of the MRI studies. Since the influencing factors of different types of treatments were not assessed in detail; this aspect needs to be refined and improved in future research. Finally, this study did not evaluate anxiety, depression, and hearing function in patients with T2DM, and hence, it would be beneficial for future research to focus on the correlation between these aspects and cognitive function.

## Conclusion

In conclusion, this study used FC and graph theory methods and showed that in patients with T2DM, multiple brain networks function abnormally and corroborate each other. In those patients, a loss of long connections is observed between the bilateral primary auditory cortices, and the functional disconnectivity between the right medial frontal cortex of the salient/ventral attention network and the left primary auditory cortex of the sensorimotor network was related to MoCA scores and blood glucose levels. These results indicate that abnormalities in the auditory cortex in patients with T2DM might be linked to cognitive impairment, providing a new perspective on the neural mechanism of cognitive impairment in T2DM, needing to be further investigated in future studies. In terms of the classification prediction of patients with T2DM and HC using SVM, the nodal degree centrality of the PCC was found to be a characteristic variable of the classification model; therefore this might serve as a potential neuroimaging biomarker of brain injury in T2DM.

## Data availability statement

The raw data supporting the conclusions of this article will be made available by the authors, without undue reservation.

## Ethics statement

The studies involving human participants were reviewed and approved by the Ethics Committee of Affiliated Zhongshan Hospital of Dalian University. The patients/participants provided their written informed consent to participate in this study.

## Author contributions

LL carried out the data collection and interpretation, and drafted the initial article. JZ carried out the data analysis and drafted the data processing part of the article. XH carried out the data analysis of SVM and drafted the SVM part of the article. YL and HX participated in the statistical analysis and drafted this section. JS and YY joined in the data collection. FC contributed to the conception and design of the study. JW and HL contributed to the conception and design of the study, interpretation of data, and manuscript revision. All authors read the final manuscript and approved it for publication.

## Footnotes

1
https://github.com/ThomasYeoLab/CBIG/tree/master/stable_projects/brain_parcellation/Schaefer2018_LocalGlobal

2
https://www.nitrc.org/projects/gretna/
