# Supplementary material for: Aberrant brain functional networks in type 2 diabetes mellitus: A graph theoretical and support-vector machine approach
Source: Front Hum Neurosci. 2022 Oct 12;16:974094. doi: 10.3389/fnhum.2022.974094 (PMC9597867; doi:10.3389/fnhum.2022.974094)
Supplement: Supplementary file 1 [file Data_Sheet_1.docx]

Supplementary Material

# Supplementary Data

The partial correlations correlation analysis were conducted between FC and clinical/cognitive scores, the threshold was set to P < 0.05, Bonferroni correction was used to control the multiple comparisons. Bonferroni correction was applied to compare the differences of topological metrics between T2DM and HC; the threshold was set to P < 0.05.

Fig.1A shows the MoCA was positively correlated with FC of right frontal medial_1 and left S2_2 for all participants (*r =* 0.2041, *P* = 0.0232). Fig. 1B shows the MoCA was positively correlated with FC of right frontal medial_1 and left auditory cortex_1 for all participants (*r* = 0.1961, *P* = 0.0271). Fig.1C shows the glucose was negatively correlated with FC of right frontal medial_1 and left auditory cortex_1 for all participants (*r* = -0.2119, *P* = 0.0218). However, when Bonferroni correction was performed for multiple comparisons, no correlation survived for Figs.1A-1C. Fig.1D shows glucose levels were significantly negatively correlated with FC of left S2_1-right TemPar_1 for all participants (*r* = -0.2590, *P*= 0.0048, Bonferroni corrected *P* < 0.05). Without the influence of healthy control, the correlation levels between FC and MoCA/glucose for T2DM group were no more significant, and there were no correlations between FC and MoCA in T2DM; this might be because T2DM’s cognitive function was not significant impaired, and it was not linear enough with the changed FC brain regions; second, MoCA is an assessment tool for rapid screening of cognitive dysfunction, including attention, executive function, memory and so on, but also affected by education level and cultural backgrounds, so such comprehensive cognitive scales which may not fully reflect subtle and refined changes of T2DM on memory and attention. We need more effective and refined cognitive rating scales for a particular cognitive domain. Zhang et al. (2016) also did not find any significant associated between fMRI indices and blood glucose levels, they thought the lack of correlation may indicate changes in activation and functional coupling of working memory networks are directly associated with neuronal impairment of working memory network rather than with blood glucose level. Cui et al. (2015) thought that the hyperglycemia may not be the most important contributor for the cognitive important. Thus, whether hyperglycemia is an independent risk factor for impaired cognition is still under debate, the mechanisms underlying changes in the brain activation during hyperglycemia are not clear. In our study, our main conclusions are not related with the relationship between FC and MoCA/glucose, thus, we put this part to supplementary materials.

We calculated the average of the correlation matrixes of all subjects after taking the absolute values with different thresholds (threshold |r| > 0.85, 0.8, 0.7 and 0.6) to obtain the average brain matrix and comparison map.

Fig.2 Long-range connectivity between the bilateral auditory cortexes is missing in patients with T2DM. In addition, the connectivity of the visual network, sensormotor network, dorsal attention network, connectivity between right dorsal attention and control network are missing or weaker in the threshold |r| >0.8.

Fig.3 Long-range connectivity between the bilateral auditory cortexes is weaker in patients with T2DM, in addition to the connectivity of the visual network, sensormotor network, dorsal/ventral attention network, control network, and default mode network are missing or weaker in the threshold |r| >0.7.

Fig.4 Long-range connectivity between the bilateral auditory cortexes is weaker in patients with T2DM; as the threshold decreases, multi-network connectivity is missing or weaker.

# Supplementary Figures and Tables

## Supplementary Figures


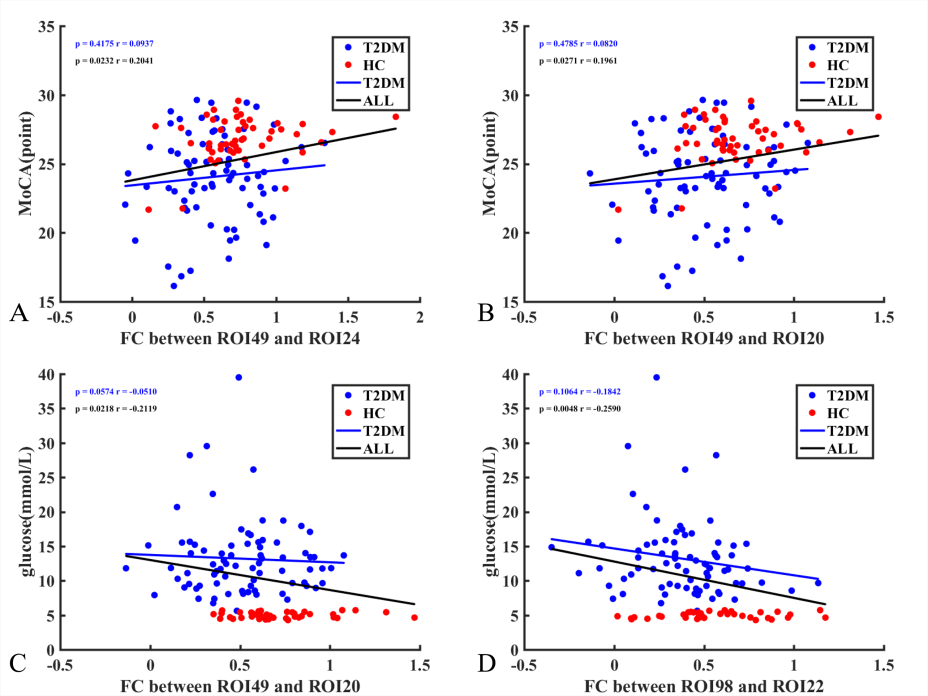


Fig. 1 Correlations between the functional connectivity and MoCA scores and fasting blood glucose in T2DM.


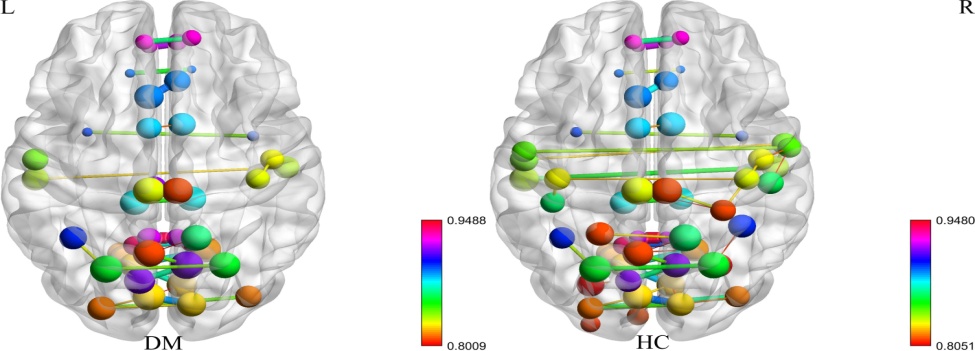


Fig.2 Average connectivity in the T2DM and HC groups in the thresholds (|r| > 0.8)


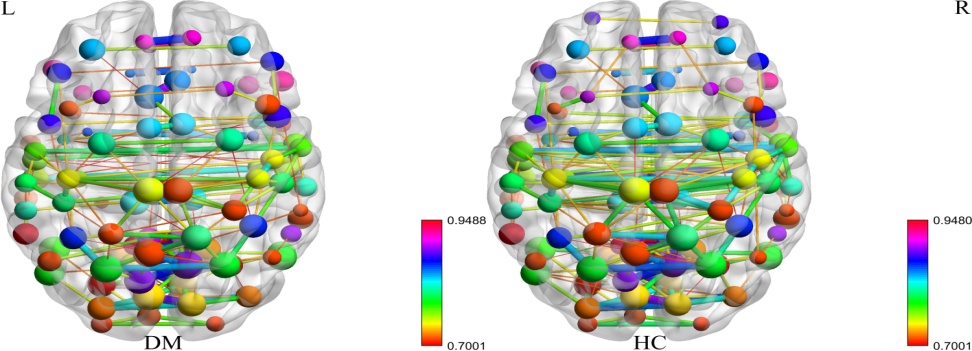


Fig.3 Average connectivity in the T2DM and HC groups in the thresholds (|r| > 0.7)


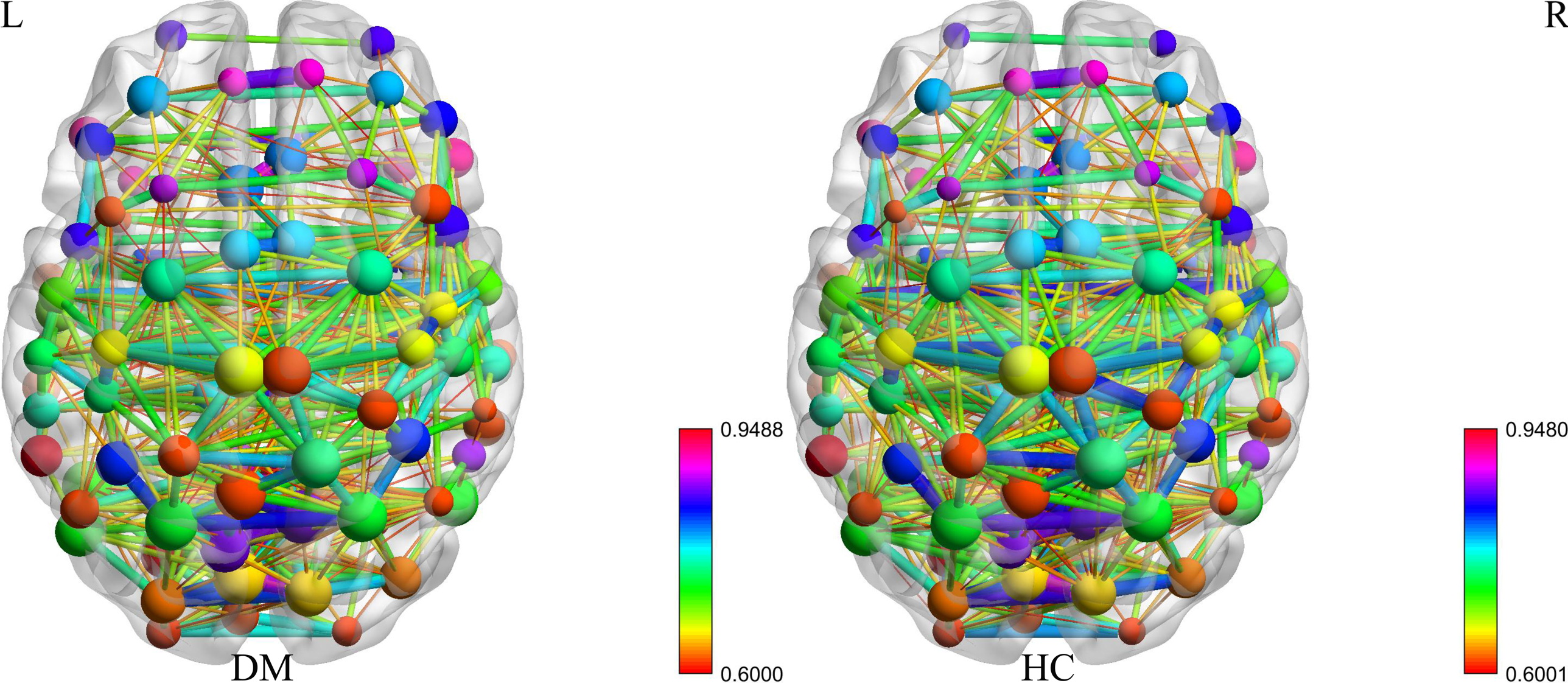


Fig.4 Average connectivity in the T2DM and HC groups in the thresholds (|r| > 0.6)

References

Cui, Y., Jiao, Y., Ding, J., Luo, B., Ju, S.H., Teng, G.J., et al. (2015) Aberrant functional connectivity of default-mode network in type 2 diabetes patients. Eur Radiol, 25; 3238-3246. doi: 10.1007/s00330-015-3746-8.

Li, Y.F., Liang, Y., Tan, X., Chen, Y.N., Ma, X. M., Qiu, S.J., et al. (2020). Altered functional hubs and connectivity in type 2 diabetes mellitus without middle cognitive impairment. Front Neurol, 11; 11:1016. doi: 10.3389/fneur.2020.01016.

Zhang, Y., Lu, S., Liu, C.L., Ni, C.L., Qin, W., Zhang, Q., et al. (2016). Altered brain activation and functional connectivity in working memory related network in patients with type 2 diabetes: An ICA-based analysis. Sci Rep, 6; 23767. doi: 10.1038/srep23767.
